# Postoperative pain following root canal treatment with XP-Endo Finisher–assisted irrigant activation: a double-blind randomized controlled trial

**DOI:** 10.1186/s12903-025-07024-9

**Published:** 2025-11-12

**Authors:** Namith Rai, Raj Kumar Narkedamalli, Nidambur Vasudev Ballal

**Affiliations:** Department of Conservative Dentistry and Endodontics, Manipal College of Dental Sciences, Manipal Academy of Higher Education (MAHE), Manipal, Karnataka India

**Keywords:** XP-Endo finisher, Root canal irrigation, Postoperative pain, Irrigant agitation, Randomized clinical trial, Endodontics, Irreversible pulpitis, Pain management

## Abstract

**Background:**

Postoperative pain is a common occurrence following root canal treatment, with reported incidence ranging from 3% to 58%. Contributing factors include insufficient canal debridement, extrusion of debris, and limitations in irrigation. While mechanical instrumentation aids in reducing microbial load, effective irrigation is essential for cleaning anatomically complex areas. The XP-Endo Finisher (XPF) is an innovative instrument designed to enhance irrigation efficacy. This study aimed to compare postoperative pain outcomes following final irrigation with either the XP-Endo Finisher or conventional needle irrigation in patients diagnosed with symptomatic irreversible pulpitis.

**Methods:**

A double-blind randomized controlled trial was conducted on 80 systemically healthy patients aged 18 years and above, presenting with symptomatic irreversible pulpitis and preoperative pain scores ≤ 3 on the Numerical Rating Scale (NRS-11). Participants were randomly assigned to two equal groups: final irrigation using either the XP-Endo Finisher or traditional needle irrigation. Patients with recent analgesic use, non-restorable teeth, or known NSAID allergies were excluded. Root canal therapy was completed in two visits. Postoperative pain levels were recorded at 6 h, 12 h, 24 h, and daily up to 7 days using the NRS-11. Statistical analysis involved descriptive statistics, normality checks, and non-parametric tests for group comparisons.

**Results:**

At 6 h, the difference in pain scores between the groups was not statistically significant (XP-Endo Finisher: 2.40 ± 1.77; Needle: 1.73 ± 1.52; *p* = 0.090). However, significantly lower pain was reported in the XP-Endo Finisher group at 12 h (*p* = 0.044) and on day 2 (*p* = 0.027). No significant differences were observed from day 3 to day 7. Analgesic intake was comparable across both groups (*p* > 0.05).

**Conclusions:**

The XP-Endo Finisher resulted in reduced early postoperative pain compared to needle irrigation at specific time points, although both methods showed similar outcomes in long-term pain resolution and analgesic consumption. Improved irrigant activation may influence short-term postoperative comfort.

**Trial registration:**

This trial was retrospectively registered with the Kasturba Hospital Institutional Ethics Committee (KH IEC) under registration number 860/2020, dated 19/03/2021. It was also registered in the Clinical Trials Registry - India (CTRI) under registration number CTRI/2021/04/032667, dated 08/04/2021.

**Supplementary Information:**

The online version contains supplementary material available at 10.1186/s12903-025-07024-9.

## Background

Pain is defined as an unpleasant sensory and emotional experience associated with actual or potential tissue injury [1, 2]. Despite the efficacy of root canal therapy in mitigating pain, the emergence of postoperative discomfort following such procedures remains a prevalent issue [3] with reported incidence rates spanning from 3% to 58% [4, 5]. The etiology of postoperative discomfort following root canal intervention is multifaceted, possibly stemming from inadequacies in canal cleaning and shaping, presence of periapical pathosis, debris extrusion, canal omission, over-instrumentation, and irrigation or medicament extrusions [6–8]. Notably, there exists a strong association between preoperative and postoperative pain levels, with patients experiencing severe preoperative pain are more prone to endure exaggerated postoperative discomfort [9, 10].

The primary objective of root canal therapy is to eradicate microbial populations within the infected root canal system to levels conducive to healing [11]. While mechanical instrumentation effectively reduces microbial counts [12], it proves insufficient in completely eradicating microbes residing within accessory canals, isthmuses, and dentinal tubules [13]. Research has shown that a significant portion, ranging from 35% to 53%, of root canal walls remain unaffected by mechanical instrumentation [14]. Consequently, irrigation emerges as a pivotal component in eliminating microbes entrenched within the complexities of the root canal system.

To avoid periapical tissue damage and reduce postoperative pain, the development of a reliable irrigation delivery system is essential. While conventional irrigation utilizing a syringe and needle remains a prevalent practice, its limitations are increasingly evident. Studies have revealed its inefficacy in adequately cleansing challenging areas such as the apical third of the canal and isthmus regions [15]. In addition, the usage of needle irrigation creates an apical stagnation zone, limiting irrigant exchange beyond the needle tip and consequently diminishing its efficacy [16]. Various irrigation activation methods, like as manual agitation, acoustic or ultrasonic activation, and negative pressure systems, have been proposed to improve irrigant mobility inside the canal, hence increasing their effectiveness in the root canal system [16–18].

A novel instrument, the XP-Endo Finisher (XPF; FKG Dentaire SA, La Chaux-de-Fonds, Switzerland), has been introduced to optimize the efficacy of final irrigation protocols (FKG Dentaire SA). Crafted from NiTi MaxWire alloy (Martensite-Austentite Electropolish-Flex, FKG), this instrument exhibits a unique characteristic of shape transformation in response to varying temperature levels. This intrinsic characteristic allegedly provides enhanced flexibility, enabling three-dimensional adaptation to the canal morphology and effectively cleaning anomalies within the root canal system while preserving dentine integrity. Prior investigations have documented its efficacy in eliminating accumulated hard tissue debris, smear layer, and microbial contaminants from the root canal system [19–23].

Studies have indicated that various irrigation devices can influence the apical extrusion of debris and irrigants, potentially exacerbating postoperative discomfort [23, 24]. The XP-Endo Finisher, owing to its unique adaptive motion and flexibility at body temperature, has been shown to enhance canal cleanliness by contacting previously untouched canal walls. However, this action may also facilitate apical extrusion of debris, which could contribute to postoperative pain. El Wazan et al., (2019), in an in vitro study, demonstrated that the XP-Endo Finisher extruded a greater quantity of debris apically compared to conventional irrigation methods, suggesting a possible mechanism for increased postoperative discomfort in clinical use [25]. More recently, Ali et al. (2023) conducted a randomized controlled clinical trial comparing XP-Endo Finisher, passive ultrasonic irrigation, and needle irrigation in symptomatic irreversible pulpitis cases [26]. Their findings indicated no statistically significant differences among the groups with respect to postoperative pain levels or analgesic intake, highlighting that while XP-Endo Finisher may alter debris extrusion dynamics, its overall clinical impact on pain outcomes appears comparable to traditional and ultrasonic techniques. Taken together, these findings suggest that although the XP-Endo Finisher may theoretically increase the risk of debris extrusion, current clinical evidence does not indicate a substantial increase in postoperative pain. Nonetheless, the number of clinical studies evaluating this instrument remains limited, and further high-quality randomized trials are required to confirm these observations. Hence, the aim of the study is to compare the postoperative pain levels associated with root canal treatment in teeth with symptomatic irreversible pulpitis when using the XP-Endo Finisher for irrigant activation versus conventional needle irrigation. The null hypothesis is that there is no significant difference in postoperative pain levels between patients undergoing root canal treatment with irrigant activation using the XP-Endo Finisher and those receiving conventional needle irrigation.

**Fig. 1:**
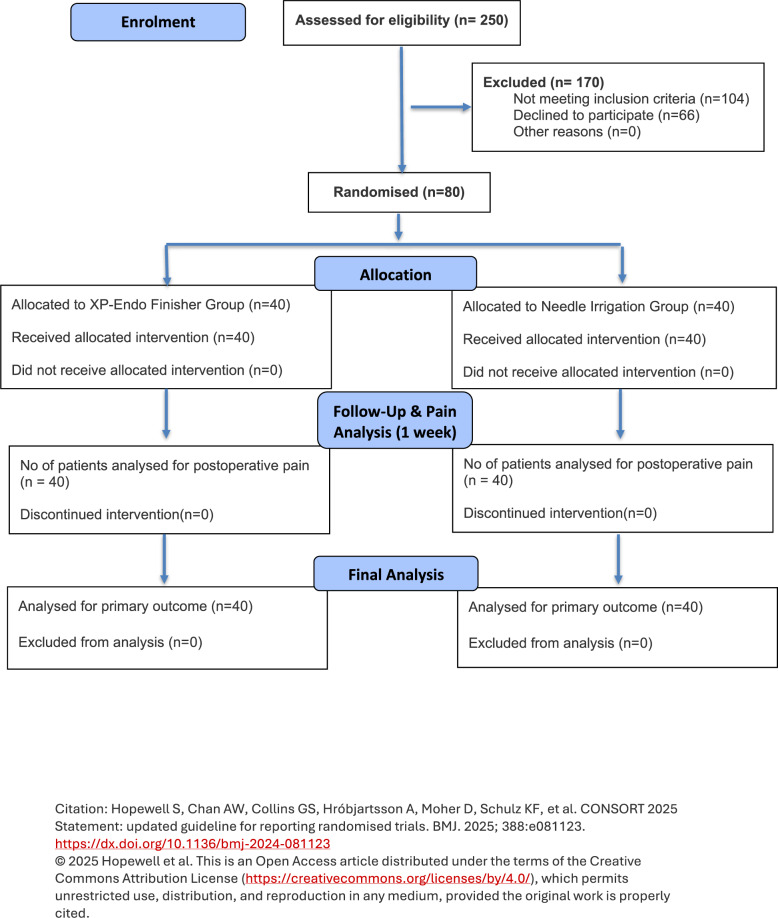
The Consolidated Standards of Reporting Trials (CONSORT) flow diagram of the patients included in this study

## Methods

### Ethics approval

The anticipated rates of postoperative pain following needle irrigation with sodium hypochlorite, as documented in previous studies, was the basis for the selection of participants for this trial (Gondim et al., 2010). This randomized controlled trial was conducted at the Department of Conservative Dentistry & Endodontics, Manipal College of Dental Sciences, Manipal, India. Ethical approval was obtained from the Kasturba Hospital Institutional Ethics Committee, Manipal, India (Ref No. 860/2020), and the study was registered prospectively in the Clinical Trials Registry - India (CTRI/2021/04/032667). The trial was designed and reported in accordance with the CONSORT 2025 guidelines for randomized controlled trials. The required sample size was calculated to be 80 patients using the formula: 

$$\mathrm n=2\left(\mathrm{Za}/2+Z\beta\right)^2\;\mathrm\sigma^2/\bigtriangleup^2$$

where α was set at 0.05, power at 90% $\left(Z\beta=1.28\right)$, the expected mean difference (Δ) in pain scores was 1.0, and the standard deviation (σ) was assumed to be 1.2, based on previous literature. Pain at 12 h post-treatment was selected as the primary outcome variable for the sample size estimation. An additional 10% was factored into account for possible dropouts. Considering these parameters, the final sample size was determined to be 80 patients, with 40 participants in each group. Accordingly, the final sample size was fixed at 40 participants in each group. A CONSORT-compliant flowchart outlining the study protocol is presented in Fig. 1.

### Eligibility criteria

The study included systemically healthy patients over 18 years of age presenting with symptomatic irreversible pulpitis in any tooth, provided their preoperative pain score on the NRS-11 scale did not exceed 3. Patients with higher baseline pain levels were excluded to minimize the likelihood of confounding due to rescue analgesic intake and to ensure ethical management, as withholding adequate pain relief in such cases would not have been appropriate. Patients were excluded if they were unwilling or unable to provide informed consent, had taken analgesic or anti-inflammatory drugs within the last 12 h, or had non-restorable teeth. Additional exclusion criteria included teeth with radiographically visible calcified canals, open or immature apices, root resorption, or tenderness to percussion. Patients allergic to non-steroidal anti-inflammatory drugs (NSAIDs), pregnant or lactating individuals, and those with periodontally compromised teeth (loss of attachment) were also excluded from the study.

The diagnosis of symptomatic irreversible pulpitis with or without associated apical periodontitis was established based on the patient’s history and clinical examination, which included palpation, tenderness to percussion, pulpal sensibility testing (cold test and EPT), and radiographic examination. A positive cold test with lingering pain and/or an EPT response of < 80 was considered diagnostic of irreversible pulpitis, whereas an EPT reading > 80 was recorded as non-vital. Prior to treatment, the severity of their current pain was measured using a numerical rating scale (NRS-11, WGMC Centre, 2003), with “0” denoting no pain and “11” denoting the most severe pain probable. Patients were thoroughly informed about the required treatment, and their participation in the study was voluntary, with each patient providing written consent. The block randomization technique was employed with a block size of 4 and a 1:1 allocation ratio (computer generated). Allocation concealment was ensured by an independent researcher not involved in this study, using sequentially numbered opaque sealed envelopes (SNOSE) containing instructions to use either needle irrigation or the XP Endo Finisher system. Subsequently, 80 patients were randomly assigned to two groups based on the method used for final irrigation after root canal preparation. Due to the distinct nature of the irrigation activation devices being investigated, blinding of the operator was not possible. Nevertheless, both the patient and the assessor analyzing the data of post-operative pain intensity at various time intervals were blinded.

### Treatment procedure

All root canal treatment (RCT) procedures were conducted within two visits by an experienced endodontist with > 5 years of clinical and academic experience. Teeth were anesthetized using a local anesthetic solution containing 2% Lidocaine Hydrochloride with 1:80,000 epinephrine (Neon Laboratories Ltd. Mumbai, Maharashtra, India). Rubber dam isolation was employed, and access cavities were prepared using high-speed burs (Dentsply Maillefer, Ballaigues, Switzerland). Pulp vitality was visually confirmed by observing bleeding upon entry into the pulp chamber. In all 80 randomized cases, pulp bleeding was observed at access preparation, thereby confirming inclusion based on pulpal status. The working length (WL) to the apical constriction was determined using an electronic apex locator (Root ZX Mini, J Morita Corp. Kyoto, Japan) in conjunction with periapical radiographs. A glide path was established with K-files up to size #15, followed by instrumentation of the canals using nickel-titanium rotary files (ProTaper Next, Dentsply Sirona, Charlotte, USA) up to size 25.06 taper. Throughout the instrumentation process, canals were irrigated with 5 mL of 3% sodium hypochlorite (NaOCl) solution (Chlor-XTRA, Vista Apex, Racine, WI) for one minute after each instrument change. Irrigation was administered using a 27-gauge side-vented needle, positioned 1 mm short of the working length (Appli-Vac, Vista Dental). The established working length was periodically reassessed during the procedure. The Final irrigation procedure after the biomechanical preparation of the root canal was categorized into two groups: conventional needle irrigation and XP Endo finisher. In conventional needle irrigation, final irrigation was carried out with 5 mL of 3% NaOCl (Vista Apex) using a syringe and a 27-gauge side-vented needle (Vista Dental) positioned 1 mm short of the working length for 2 min. Subsequently, the canals were irrigated with 5 mL of saline for 1 min, followed by 5 mL of 17% EDTA (Vista Dental) for 1 min. Finally, the canals were rinsed with 5 mL of saline solution for 1 min and dried with paper points (Dentsply Maillefer). In XP-Endo finisher group, root canals were irrigated with 2.5 mL of 3% NaOCl (Vista-Apex) for 30 s, followed by activation of the XP Endo Finisher file for 30 s. This procedure was repeated once more, resulting in a total irrigation and activation time of 1 min with 3% NaOCl. Subsequently, the canals were irrigated with 5 mL of saline for 1 min. Then the canals were irrigated with 2.5 mL of 17% EDTA (Vista Dental) for 30 s, followed by activation of the XP Endo Finisher file for 30 s. This process was repeated once more, resulting in a total irrigation and activation time of 1 min with 17% EDTA (Vista Dental). The canals were subsequently flushed with 5 mL of saline solution for 1 min and dried with paper points (Dentsply Maillefer). The access cavities were temporarily sealed with a sterile cotton pellet and Cavit (3 M ESPE, St. Paul, Minn, USA). No intracanal medicament (ICM) was placed after the initial instrumentation and irrigation procedures. The rationale for omitting ICM was that its placement could potentially reduce postoperative pain levels, thereby introducing a confounding factor. Since the primary aim of the study was to evaluate postoperative pain following different final irrigation methods, excluding ICM ensured that pain outcomes could be attributed solely to the irrigation technique used. No dropouts occurred during the trial, resulting in 100% retention of all 80 patients across both groups.

Patients were recalled after one week, at which time root canal filling was performed with gutta-percha points (Dentsply Sirona) and an AH Plus epoxy resin-based sealer (Dentsply, DeTrey, Konstanz, Germany) using the cold lateral compaction technique. Radiographs were taken at two different angulations to ensure the quality of the root filling. Post-endodontic restoration was completed, and the occlusion was checked and adjusted if necessary. Patients were advised not to take any analgesics or anti-inflammatory drugs unless they experienced pain, in which case they were instructed to take oral NSAIDs (Ibuprofen 400 mg) as and when required. Participants were instructed to contact the principal investigator if the analgesic protocol failed to provide pain relief.

### Post-treatment pain evaluation

All participants received a sheet containing the numerical rating scale (NRS-11), with “0” representing no pain and “10” indicating the worst pain experienced after shaping and cleaning of root canals. Patients were provided with a chart to document the intensity of pain and the frequency of analgesic intake at 6 h, 12 h, 24 h, 2nd day, 3rd day, 4th day, 5th day, 6th day, and 7th day following shaping and cleaning of root canals by an assessor who was blinded to the experimental groups used. All 80 patients completed their pain diaries in full, and data completeness was verified at the recall visit. No missing entries were recorded. All 80 randomized patients completed the study, and therefore the intention-to-treat (ITT) and per-protocol (PP) populations were identical.

### Statistical analysis

The statistical tests were conducted using SPSS version 28 (IBM Corp. Released 2011. IBM SPSS Statistics for Windows, Version 20.0. Armonk, NY: IBM Corp) to analyse the data, including descriptive statistics to summarize pretreatment and postoperative pain levels. The normality of data was tested using Kolmogorov-Smirnov and Shapiro-Wilk tests. Kruskal-Wallis test was used to compare pain levels between the XP-Endo Finisher and needle irrigation groups. Additionally, the Wilcoxon Signed-Rank test was used to evaluate differences in pain levels over time, and the Friedman test was applied to compare pain levels across multiple time points. Chi-square tests were conducted to examine the association between age groups, gender, and treatment groups.

## Results

Over a span of 16 months, patient recruitment and all treatment procedures were finalized. Throughout the study duration, approximately 250 patients were pre-screened by the investigators. Out of this initial pool, 80 patients who fulfilled the inclusion criteria were enrolled (Fig. 1). Table 1 presented the distribution of age groups among the needle irrigation and XP Endo Finisher groups, comprising 80 participants in total. The chi-square test (X² = 2.63, *p* = 0.854) revealed no significant difference in age distribution between the needle irrigation and XP Endo Finisher groups. Overall, 55% (44 out of 80) of the participants were female, while 45% (36 out of 80) were male. The chi-square test (X² = 0.808, *p* = 0.369) indicated that there was no statistically significant difference in demographics between the two groups.

**Table 1:** Demographic characteristics and pain levels in patients treated with XP-Endo finisher and needle irrigation (* - Significant)

Parameters	Category	XP-Endo Finisher (*n* = 40)	Needle Irrigation (*n* = 40)	Total (*n* = 80)	*p*-value
	Mean ± SD	45.06 ± 17.73	45.06 ± 17.73	45.06 ± 17.73	-
Age (Years)	≤ 20	5 (12.5%)	4 (10.0%)	9 (11.3%)	0.854
	21–30	6 (15.0%)	8 (20.0%)	14 (17.5%)	
	31–40	4 (10.0%)	5 (12.5%)	9 (11.3%)	
	41–50	8 (20.0%)	5 (12.5%)	13 (16.3%)	
	51–60	8 (20.0%)	7 (17.5%)	15 (18.8%)	
	61–70	7 (17.5%)	6 (15.0%)	13 (16.3%)	
	> 70	2 (5.0%)	5 (12.5%)	7 (8.8%)	
Gender	Female	20 (50.0%)	24 (60.0%)	44 (55.0%)	0.369
	Male	20 (50.0%)	16 (40.0%)	36 (45.0%)	
Pretreatment Pain Level	Mean ± SD	2.68 ± 0.47	2.50 ± 0.64	2.59 ± 0.57	0.258
Postoperative Pain Levels					
6 h	Mean ± SD	2.40 ± 1.77	1.73 ± 1.52	2.06 ± 1.67	0.090
12 h	Mean ± SD	1.68 ± 1.47	1.08 ± 1.35	1.38 ± 1.44	0.044*
24 h	Mean ± SD	1.25 ± 1.32	0.68 ± 1.07	0.96 ± 1.23	0.027*
2 days	Mean ± SD	0.58 ± 1.08	0.33 ± 0.76	0.45 ± 0.94	0.207
3 days	Mean ± SD	0.28 ± 0.93	0.10 ± 0.38	0.19 ± 0.71	0.443
4 days	Mean ± SD	0.05 ± 0.22	0.00 ± 0.00	0.03 ± 0.16	0.155

At the 6-hour postoperative mark, the mean pain score was 2.400 for the XP Endo Finisher group and 1.725 for the needle irrigation group. Statistical analysis using the Z-test did not show a statistically significant difference between the groups (*p* = 0.090). After 12 h, the mean pain score was 1.675 for the XP Endo Finisher group and 1.075 for the needle irrigation group. The Z-test revealed a significant difference between the groups (*p* = 0.044). At the 2-day postoperative assessment, the mean pain score was 1.250 for the XP Endo Finisher group and 0.675 for the needle irrigation group. The Z-test indicated a significant difference between the groups (*p* = 0.027). At 3, 4, 5, 6, and 7 days postoperatively, no significant differences were observed between the groups (all *p* > 0.05).

The chi-square test (X² = 0.894, df = 1, *p* = 0.344) indicated that there was no statistically significant difference in analgesic consumption between the XP Endo Finisher and needle irrigation groups (16/40 vs. 11/40 patients, respectively). These data are summarized in Table 2.

**Table 2:** Analgesic consumption by group

Group	Took Analgesics	Did not take Analgesics
XP Endo Finisher (*n* = 40)	16	24
Needle (*n* = 40)	11	29

## Discussion

The extrusion of irrigants and debris beyond the apical foramen into periapical tissues is a critical factor that can lead to postoperative discomfort and pain. This phenomenon is largely due to the inflammatory response triggered by foreign materials and chemicals in sensitive periapical tissues [27]. Studies have shown that minimizing this extrusion is vital for improving patient outcomes and comfort following endodontic treatments [28].

Postoperative pain is a highly individual experience, influenced by numerous factors such as the patient’s pain threshold, psychological state, type and extent of surgery, and even genetic predispositions. The subjective nature of pain poses significant challenges for accurate measurement and assessment. To address these challenges, researchers and clinicians have employed a range of scales and methods designed to capture the variations of postoperative pain. These tools include numerical rating scales (NRS), visual analog scales (VAS), and more comprehensive assessments like the McGill Pain Questionnaire (MPQ), each tailored to quantify pain intensity, quality, and impact on the patient’s daily life [29]. Utilizing these diverse methodologies ensures a more holistic understanding and management of postoperative pain, ultimately leading to better patient outcomes and enhanced pain management strategies. The NRS (Numeric Rating Scale) is a valuable tool in dentistry for assessing pain intensity on a scale of 0 to 11, where 0 represents no pain and 11 represents the worst imaginable pain. Its widespread adoption stems from its straightforwardness and efficacy in appraising dental pain, supporting clinicians in treatment decisions and patient care. McGrath et al., (2004) found that patients prefer the NRS due to its user-friendly nature and accuracy in communicating pain levels, particularly vital in dental contexts where precise pain management is critical [30].

The research limitations in assessing post operative pain pertain to the challenges and variances in study designs, including the preoperative state of the tooth and treatment techniques [31]. The current study excluded teeth identified with apical periodontitis and pulp necrosis, while adhering to a meticulous aseptic protocol to minimise the possibility of pain exacerbation from remaining microorganisms or bacterial contamination. Therefore, only teeth diagnosed with irreversible pulpitis were subjected to intervention. The current study included only teeth presenting with a single canal in the pre-operative radiographic examination to decrease the possibility of iatrogenic errors due to undetected or complex root canal anatomy and also to ensure uniform distribution of the irrigant throughout the root canal. All the teeth were treated and filled in in two separate appointments. This was done to assess the postoperative pain levels in patients after the first session. Following the preliminary cleaning and shaping of the root canals, no intracanal medicament was placed in the canals to alleviate any possible reduction of postoperative pain levels. Moreover, only patients devoid of a relevant medical history and who had not recently received analgesic medication were included to eliminate any potential confounding factors related to pain sources or drug interactions affecting the pain following endodontic therapy.

The current study adequately recruited 80 patients over a 16-month duration, with participants randomly assigned to two groups of 40 patients each, thus strengthening the internal validity of the research. The baseline demographic study indicated that the groups were comparable for age and gender distribution, demonstrated by non-significant chi-square test results. The demographics were not significantly different between the groups, which minimizes the potential for bias related to age or gender differences in pain perception and analgesic response.

In the present study, postoperative pain evaluation indicated that, at the 6-hour mark, the average pain scores for the XP Finisher and Needle groups were 2.400 and 1.725, respectively. The XP Finisher group demonstrated elevated pain scores; however, the difference was not statistically significant (*p* = 0.090), indicating comparable early postoperative pain levels across the two groups. At 12 h, the XP Finisher group had markedly decreased pain levels (*p* = 0.044), suggesting a more favourable tendency in pain reduction compared to the needle group. At the 2-day postoperative interval, the XP Finisher group exhibited markedly decreased pain scores compared to the needle group (*p* = 0.027). Although these differences reached statistical significance, the absolute mean difference in pain scores was less than 1 on the NRS scale, indicating a modest effect size with limited clinical impact. These findings indicate that while early postoperative pain management was comparable between the two groups, the XP Finisher group exhibited enhanced pain reduction over time. This could be attributed to differences in mechanical debridement efficiency and debris removal, potentially influencing postoperative inflammatory responses. Beyond the 2-day mark, pain scores in both groups converged, with no significant differences observed at 3, 4, 5, 6, and 7 days postoperatively. This indicates that any initial disparities in pain perception were transient, and by the subsequent postoperative phase, both methods had similar clinical results. This may relate to the transient inflammatory response in the periapical tissues, with PMNs dominating in the early phase (within 6 h) and macrophage infiltration occurring after 48 h [32]. The resolution of pain over time in both groups correspond with the inherent progression of postoperative recovery, suggesting that both methods are efficacious in attaining sustained pain reduction. It should also be acknowledged that assessing pain at multiple time points may increase the likelihood of type I error; therefore, pain at 12 h was defined a priori as the primary outcome measure, while analyses at other time points were considered exploratory.

Analgesic consumption, a critical indicator of postoperative pain management, did not differ significantly between the groups (*p* > 0.05). This suggests that despite observed differences in pain scores at specific time points, the overall need for analgesic medication remained comparable between the XP Finisher and needle groups. This study illustrates the clinical significance of pain score variations, as they did not result in changes to analgesic consumption patterns, possibly suggesting that both strategies offered sufficient pain management.

The clinical implications of these findings are significant. The XP Finisher system, designed to enhance canal cleanliness through its unique adaptive motion, demonstrated superior pain reduction at 12 h and 2 days postoperatively. However, the needle group exhibited similar pain outcomes in the later postoperative period. These findings align with previous studies suggesting that mechanical agitation techniques can influence early postoperative pain levels by modulating debris extrusion and inflammatory response [27, 28, 33]. However, the absence of notable disparities in analgesic usage indicates that both methods offer clinically satisfactory postoperative relief.

## Conclusion

Both conventional needle irrigation and the XP-Endo Finisher system demonstrated effective postoperative pain management after root canal therapy. At 12 h and 2 days postoperatively, the XP-Endo Finisher demonstrated a transient advantage in pain reduction; nonetheless, both groups’ participants consumed comparable amounts of analgesics overall. The data indicate that although improved irrigant activation may affect early postoperative pain, XP-Endo Finisher reduced early postoperative pain compared to needle irrigation, though both groups showed similar long-term pain outcomes. Additional research with larger sample sizes and varied clinical situations would be beneficial to validate these findings.

## Supplementary Information

Supplementary Material 1

## Data Availability

The corresponding author can provide the datasets used and/or analyzed during the current study upon request.
